# Stable, Free-space Optical Trapping and Manipulation of Sub-micron Particles in an Integrated Microfluidic Chip

**DOI:** 10.1038/srep33842

**Published:** 2016-09-22

**Authors:** Jisu Kim, Jung H. Shin

**Affiliations:** 1 KAIST, Department of Physics, 373-1 Guseong-dong, Yuseong-Gu, Daejeon, South Korea; 2 KAIST, Graduate School of Nanoscience and Technology, 373-1 Guseong-dong, Yuseong-Gu, Daejeon, South Korea

## Abstract

We demonstrate stable, free-space optical trapping and manipulation in an integrated microfluidic chip using counter-propagating beams. An inverted ridge-type waveguide made of SU8 is cut across by an open trench. The design of the waveguide provides low propagation losses and small divergence of the trapping beam upon emergence from the facet, and the trench designed to be deeper and wider than the optical mode enables full utilization of the optical power with an automatic alignment for counter-propagating beams in a trap volume away from all surfaces. After integration with polydimethylsiloxane (PDMS) microfluidic channel for particle delivery, 0.65 μm and 1 μm diameter polystyrene beads were trapped in free space in the trench, and manipulated to an arbitrary position between the waveguides with a resolution of < 100 nm. Comparison with numerical simulations confirm stable trapping of sub-micron particles, with a 10 k_B_T threshold power of less than 1 mW and a stiffness that can be 1 order of magnitude larger than that of comparable fiber-based trapping methods.

Since the first introduction by Ashkin, optical trapping of particles has become a powerful tool in many diverse fields due to their ability to trap, manipulate, and sort micro- and nanometer sized particles, ranging from dielectric spheres and cells to viruses and DNA, without any direct physical contact^1^,^2^,^3^,^4^,^5^,^6^,^7^,^8^,^9^,^10^,^11^. The earliest, and most widely available systems are based on off-chip, free-space optical systems^12^,^13^,^14^,^15^. While they allow for a wide range of possible experimental configurations, they can be bulky, and require expensive stabilization systems and high optical powers^16^.

As an alternative, planar, integrated optical structures have attracted a great interest as a possible solution to above problems. As all elements, including non-optical devices, are defined by lithography, precise alignment of diverse elements is possible, resulting in a compact, robust, and multi-functional chip that can be mass-produced at a low cost^17^,^18^,^19^. Furthermore, such a chip can easily be integrated with microfluidics as well, for an all-in-one lab-on-a-chip system^20^,^21^.

In planar structures, evanescent field is often used for trapping since strong intensity gradient is produced near the surface of the photonic devices. While such evanescent-field based trapping allows for easy and precise transport along the waveguide^22^,^23^,^24^,^25^,^26^,^27^,^28^,^29^,^30^,^31^, it also leads to unavoidable contact with the device surface, eliminating one of the main advantages of optical trapping. Such contact can disrupt many biological processes^32^,^33^, and can even strongly deform trapped particles as well^34^. To avoid these problems, counter-propagating beam method that uses the gradient force and scattering forces from opposing beams to provide the axial and longitudinal confinement, respectively, has been proposed^35^,^36^. As it separates trapping optics from imaging optics^37^,^38^, counter-propagating beam method is well-suited for planar trapping geometry. By now, optical fibers^39^,^40^,^41^,^42^,^43^,^44^, waveguides^45^, and even direct integration of lasers^46^ have been used to successfully, demonstrating its potential to provide a platform for on-chip optical trapping and manipulation.

Still, several issue remain with the results reported so far. Fiber-based approaches remain rather bulky, and aligning the fibers can still require delicate assemblies^47^,^48^,^49^. Direct integration of laser can provide the highest level of integration, but the fabrication can be quite complex, and it sacrifices the ability to vary the wavelength, polarization, and coherence of the counter-propagating beams to control the trapping mechanism^46^. Furthermore, both direct integration of lasers and high-index waveguides result in strong beam divergence due to the large index contrast with water, which can reduce the volume and stiffness of the trap.

In this article, we report on stable, free-space optical trapping and manipulation using counter-propagating beams in an integrated microfluidic chip with inverted ridge-type waveguides made of SU8, and a microfluidic channel made of polydimethylsiloxane (PDMS). The waveguide is cut across by an open trench that is deeper and wider than the optical mode in order to provide a large trap volume away from any surfaces, automatic alignment of counter-propagating beams and full utilization of input optical power. The inverted ridge design keeps the optical mode away from the top surface of the waveguide, which not only reduces the propagation loss, but also prevents unwanted trapping by the evanescent field such that trapping occurs only inside the trench. In addition, the use of SU8 provides low refractive index contrast which reduces the divergence of the trapping beam. The vertical and horizontal divergence angles are 4.8 and 18.2 degrees, respectively, which are comparable to what have been achieved using specially designed fiber tips^44^. Finally, we demonstrate stable trapping of 0.65 μm and 1 μm diameter polystyrene beads, both a single particle and an array of multiple particles, in the free space in the trench, and their manipulation to an arbitrary position between the waveguides with a resolution of < 100 nm. Detailed numerical and analytical calculations agree well with experimental results, and indicate a strap stiff ness of 1.35 and 0.12 pN μm^−1^ mW^−1^ for longitudinal and transverse direction, with a 10 k_B_T threshold of 0.29 mW, for a single 1 μm diameter particle. These stiffness values are 4.7 times larger, at an input power that is more than 10 times lower, than those achieved by a similar optical trap with high index waveguides^50^. Indeed, these values are comparable to those obtained from fibers with specially designed tips^44^, despite the simple, planar design without any specific attempts at beam shaping.

## Results

### Formation of waveguides with an open space

**Figure 1:**
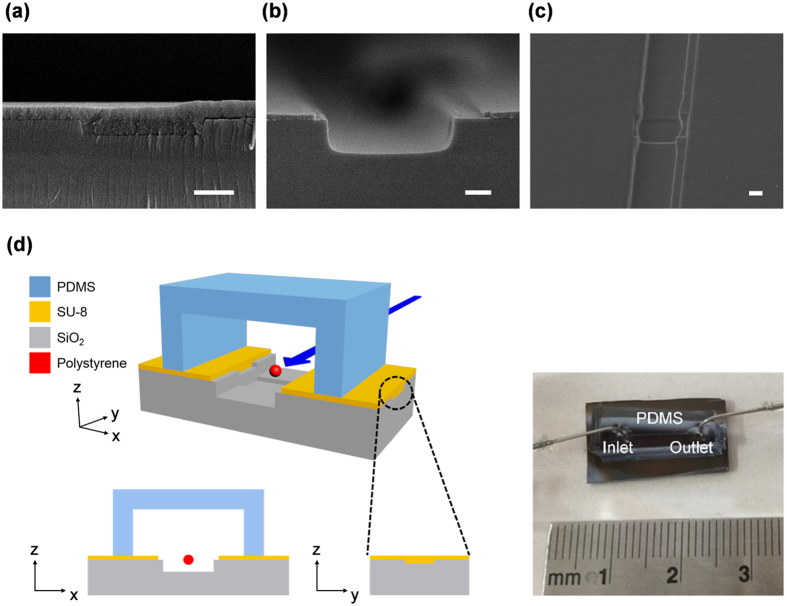
Cross-section SEM images of (**a**) the waveguide; (**b,c**) the trench for particle trapping prior to integration with the PDMS fluidic channel. The scale bar in all images are 1 μm. (**d**) A schematic illustration of the finished chip, together with a photograph of an actual chip.

To fabricate the integrated microfluidic chip, a 2.8 μm wide, 400 nm shallow trench is formed on a 10 μm thick thermal oxide wafer, followed by formation of a 4.9 μm wide, 1.7 μm deep trench perpendicular to the shallow ridge. SU8 is then spin-coasted on the entire wafer to a thickness of 420 nm, followed by a final lithographic step to expose the deep trench. The shallow ridges define an inverted-ridge waveguide, and the exposed trench provides space in which suspended particles can be trapped freely. The entire structure is then covered with a PDMS layer to form a microfluidic channel (100 μm wide, 5 μm thick) parallel to the deep trench. Finally, the chip is cleaved to induce efficient coupling of optical power into the waveguide using external optical fibers, and syringe pumps are connected to the PDMS microchannel using oxygen plasma (See supplementary Fig. S1). Figure 1a shows an SEM image of the cross-section of waveguide, showing the inverted ridge structure. Figure 1b shows an SEM image of the trench, and Fig. 1c shows a top-view SEM image of the gap and waveguides, confirming that self-aligned waveguides with the open free space for particle trapping are successfully formed. A schematic drawing of the fabricated chip, together with a photograph of an actual chip, is shown in Fig. 1d. The coupling loss and the propagation loss of the waveguides were estimated to be 5.24 dB and 1.74 dB/cm, respectively (data not shown).

### Calculated optical guided mode

**Figure 2:**
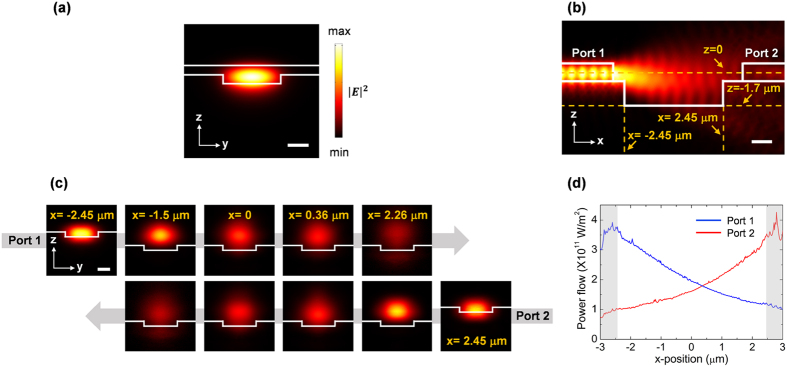
3D Finite element method (FEM) simulations of the optical modes. (**a**) Mode profile of the fundamental TM mode supported by a waveguide. (**b**) A cross-section view of |*E*|^2^ distributions, cut parallel to the waveguide. Note that the beam diverges upon emerging from the waveguide. (**c**) shows the field distribution at each given positions in the water. Mid-gap position is defined to be x = 0 and the center of the waveguide width is at y = 0. (**d**) Calculated power flow of the counter-propagating guided beams. Note that a power equilibrium position exists between two waveguides. Scale bar, 1 μm.

Figure 2 shows the optical mode of the fabricated chip, calculated by finite element method (FEM) using SEM images of the actual chip. In all cases, aqueous environment and wavelength of 1550 nm is used. As shown in Fig. 2a, the SU8-filled shallow trench forms a single-mode waveguide that supports the fundamental TM mode. Note that the inverted structure pulls the optical mode away from the exposed surface, resulting in a weak evanescent field. This not only prevents unwanted trapping of particles by the waveguide, but also reduces the absorption loss by water in the microfluidic channel. The mode overlap of evanescent field in water is calculated to be of 6.8%. Thus, given the small trapping volume and short length over which the waveguide is exposed to water, the propagation loss was not considered in numerical calculations. The transverse and cross-sectional beam profiles within the trench are shown in Fig. 2(b,c), respectively. Note that the trench is deeper than the optical mode, with waveguide facets positioned above the bottom of the trench such that the most of the input power is confined within the trench. Furthermore, even without any special shaping of the waveguide facet, the beam divergence is quite modest with vertical and horizontal divergence angles of 4.8 and 18.2 degrees, respectively, comparable to what has been achieved using specially designed fiber tips^44^. These factors, together with the compact device size, enable full utilization of the optical power with intensity in the center of the trapping trench that is reduced to only about half of its value in the waveguide unlike previous reports that used high index waveguides^50^ or semiconductor lasers^46^.

### Trapping of particles suspended in water

**Figure 3:**
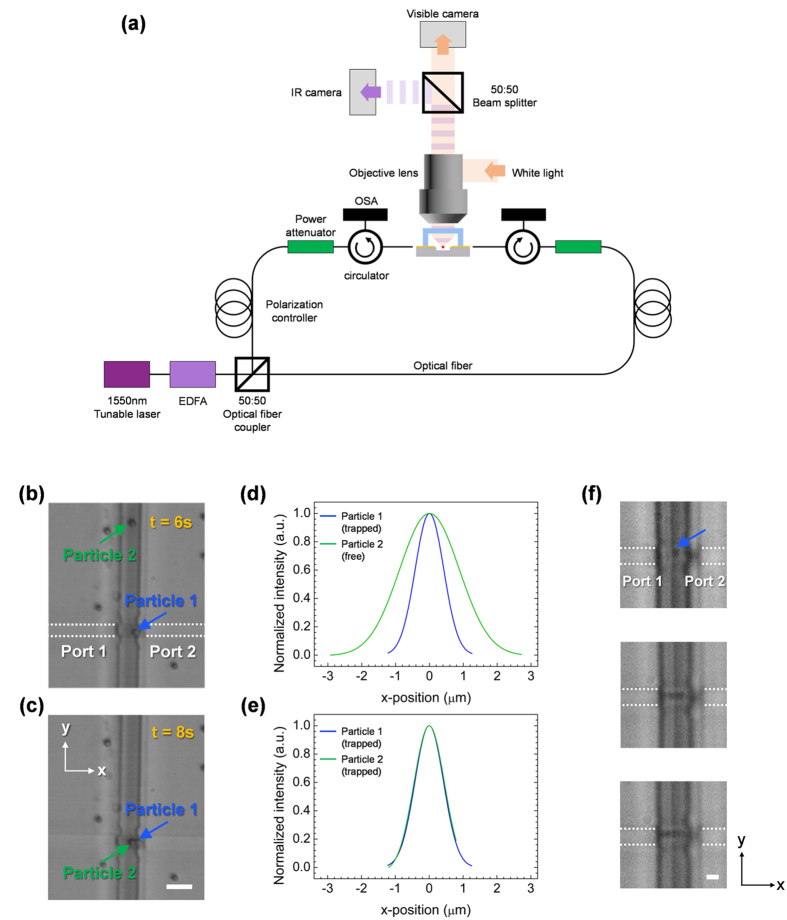
(**a**) A schematic drawing of an experimental setup. (**b**,**c**) The optical images of a trapped and a free 1 μm diameter polystyrene spheres in water (See associated media file (Movie 1). Scale bar, 5 μm). The apparent size of the particles in the image (**d**) before and (**e**) after trapping. Curves with same colors correspond to the same particles. The curves are normalized to the maximum intensity for each particle. (**f**) Single and multiple trapping of 0.65 μm diameter polystyrene particles (See associated media file (Movie 2). Scale bar, 2 μm).

Optical trapping and manipulation in the fabricated chip is demonstrated using aqueous suspension of polystyrene spheres (Corpuscular). For observation, a ×40 objective lens was used for a theoretical optical resolution of 0.71 μm (for an illumination wavelength of 0.7 μm) and a depth of field (DOF) of 2.77 μm^51^. The experimental setup for optical trapping is shown schematically in Fig. 3a. A 1550 nm laser beam is split into two beams, and coupled into the two waveguides using lensed fibers. Both input beams were controlled to be TM-polarized, and the input powers were monitored using an optical spectrum analyzer. The images of light intensity and trapping results are recorded by CCD camera (See Methods). The lowest optical power used for trapping was 7.3 mW at the input facet of the waveguide, which is estimated to correspond to 1.79 mW output for trapping, based on the coupling efficiency and the waveguide propagation loss. Figure 3b,c show the trapping results of 1 μm particles moving along the channel perpendicular to the waveguides (defined to be the y-direction. See Movie 1). A free particle first moves along the channel, and is trapped when it encounters the waveguides. Water flow was cut after particle trapping.

Multiple particles trapping is also possible. Indeed, as Fig. 3d,e show, when the focal plane of the microscope is fixed at the position of trapped particle 1, the apparent size of the yet-to-be-trapped particle 2 is much larger, indicating that its vertical position is different from that of the trapped particle^50^. Upon trapping, its apparent size changes to that of trapped particle 1 (0.99 μm, in good agreement with the actual size of the particle), indicating that it is attracted toward the position of intensity maximum, and thus suspended freely in the water, away from all surfaces. The trapping is quite stable, as a particle can remain trapped as long as the laser power is turned on, without any observable damage to the polystyrene spheres. In fact, 0.65 μm diameter spheres can also be easily trapped, as are shown in Fig. 3f. The optical resolution of the setup limits the smallest particle whose trapping can be observed reliably. However, the present setup is expected to be capable of trapping particles with diameter as small as 0.35 μm, as shall be shown later.

### Control of a position of the trapped particle

**Figure 4:**
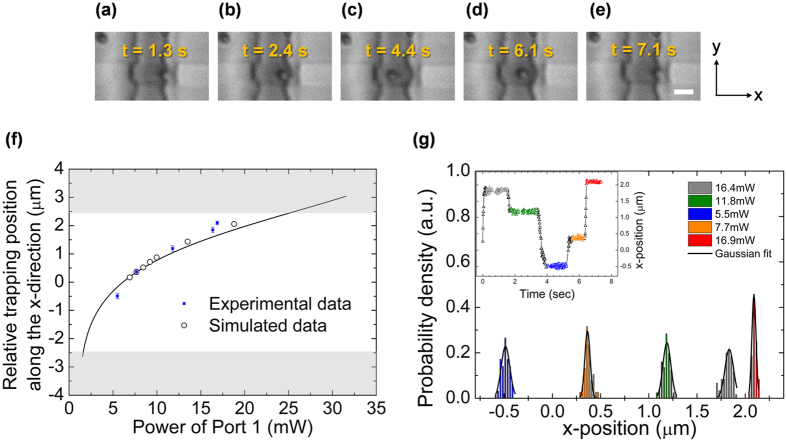
Demonstration of particle manipulation by varying the power ratio. (**a–e**) Time-lapsed images of a trapped particle in both direction. Scale bar, 2 μm. (**f**) Longitudinal trapping position for controlling the relative power of two beams. Error bar for position measurements is the FWHM of each position due to the Brownian motion as shown in (**g**). The input power at Port 2 is fixed at 7.3 mW, while the input power at port 1 was varied 5.5 mW and 16.9 mW (See Movie 3). (**g**) Results of longitudinal probability density (inset) of the trapped particle as a function of the relative distance between two waveguides. It is fitted with a Gaussian function (black lines).

We note that such counter-propagating beams can generate standing wave patterns due to interference, which can lead to strong gradient forces^52^. In our case, the difference in the beam intensity away from the equilibrium point and the relatively large size of the particle compared to the expected interference pattern ensure that the scattering force dominates the longitudinal trapping force. Such dominance of the scattering force can be confirmed by, and utilized for, manipulating the longitudinal position of the trapped particle by controlling the relative power of the counter-propagating beams^47^. Figure 4a–e show the results of such manipulation, in which we trap the particle at an arbitrary position along the x-axis between the waveguides by simply varying the power ratio using external power attenuators (See Movies 3). Furthermore, as Fig. 4f shows, the observed trapping positions agree very well with the positions calculated using scattering forces only, confirming that the trapping is dominated by light intensity, and that we can predictably place a particle at any point between the waveguides within the trench. The resolution of such particle placement is less than 100 nm across the entire trench, as shown in Fig. 4g. Such a resolution limit is poorer than those reported for evanescent-field based trapping, as these particles are suspended freely in water, and are subject to free Brownian motion. When compared to similar works on trapping suspended particles using dual beams, the resolution of <100 nm is up to 2.5 times better even though the experiment is performed at a lower power, demonstrating the advantage of the design used in the present work^50^.

### Analysis of the trapping force

**Figure 5:**
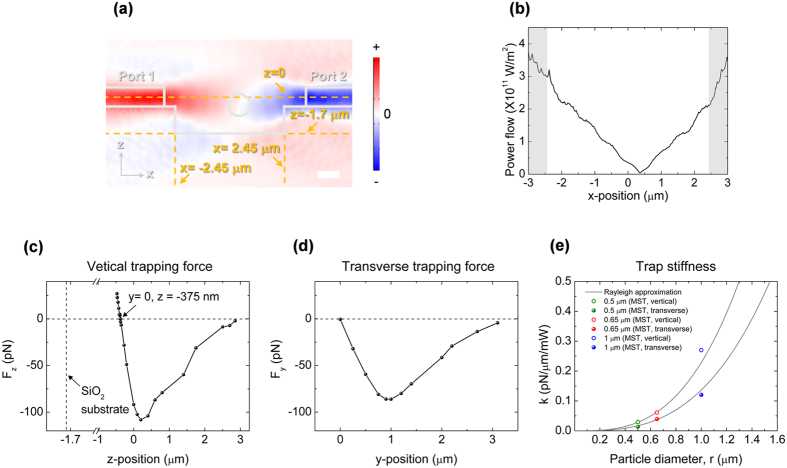
3D Finite element method (FEM) simulations of power flow and trapping force. Two counter-propagating beams are excited on each port and the each beam power of 1W is assumed. (**a**) A cross-section view of power flow, cut parallel to the waveguide. (**b**) Calculated magnitude of power flow along the x-axis at y = 0 and z = −375 nm position. Note that the position at x = 360 nm corresponds to the power equilibrium position in Fig. 2d. (**c,d**) Are vertical and transverse trapping forces, respectively, at x = 360 nm. (**e**) Shows calculated trapping stiffness along the y-axis in terms of different particle sizes. Solid dots are calculated by Maxwell stress tensor (MST) and solid gray line is a fitting curve by assuming Rayleigh particle (See Supplementary Fig. S2).

In order to quantitatively analyze the experimental results, we perform detailed calculations of the optical forces encountered in this work. As shown in Fig. 5a,b, we first calculate numerically the optical power flow in the xz plane, cut parallel to the waveguides, using the actual fabricated structures shown in Fig. 1. Along the x (longitudinal) direction, the intensity equilibrium position is found to be at x = 360 nm, with the center of the trench defined as x = 0 due to the fabrication offset (See also x-component of Poynting vector over time in Supplementary Movie S1, as calculated by finite-difference time-domain (FDTD) simulation). Based on the optical power, the axial force along the vertical and transverse directions, exerted on a 1 μm diameter polystyrene bead at the equilibrium point when two beams are of equal intensity are calculated using Maxwell stress tensor formalism^53^ as are shown in Fig. 5c,d. By symmetry, the transverse force is zero at y = 0 in the yz plane, which is defined to be the center of the waveguide. As can be seen in Fig. 5c, the vertical force is zero is at x = 360 nm, y = 0, and z = −375 nm. As the bottom of the trench is located at z = −1.7 μm, this indicates that the equilibrium position is well away from the bottom of the gap, and that the trapped particles will be suspended in space, away from the surface. By measuring the slope of the trapping force near the stable position, trap stiffness can be calculated. The calculated results for particles of different diameters are shown in Fig. 5e. Also shown for comparison is the analytical results obtained using Rayleigh approximation. We find that within the size range explored here, the Rayleigh approximation agrees quite well with the numerical Maxwell tensor results without any attempts at fitting.

The calculated trap stiffness, stability number and the threshold power (defined to be 10 k_B_T, where k_B_ is the Boltzmann constant, T is the temperature) are summarized in Table 1. Also shown for comparison is the experimentally measured transverse trap stiffness for 1 μm diameter particle, obtained by analyzing the Brownian motion of the trapped particle and analyzing its probability density function by fitting it to Gaussian distribution as following relation.


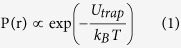


*k*_*trap*_ is trap stiffness and 
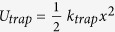
 is the potential^54^. The calculated and experimentally obtained trap stiffness values agree very well, demonstrating the validity of the methods used in calculations presented in this paper. We find that the longitudinal stiffness, caused by scattering forces of the counter-propagating beams, is much larger than the axial stiffness caused by the gradient force. Still, the stiffness value of 0.12 pN μm^−1^ mW^−1^ is nearly 1 order of magnitude larger than the values that have been previously reported from comparable particle trapping results using counter-propagating beams^44^,^50^, demonstrating the advantage of our compact, low-divergence optical design. Clearly, with higher input power, smaller particles can be trapped. For an input power of 100 mW, which corresponds to a trapping power of 24.5 mW, we estimate that particles with diameter as small as 0.42 μm can be stably trapped. In our setup, the maximum input power is 170 mW, which would enable trapping of 0.35 μm diameter particle. We note, however, that this limit can be reduced further by better fabrication methods to reduce coupling and propagation losses, and with better facet designs to reduce the beam divergence even further^40^.

### Multiple particle trapping

**Figure 6:**
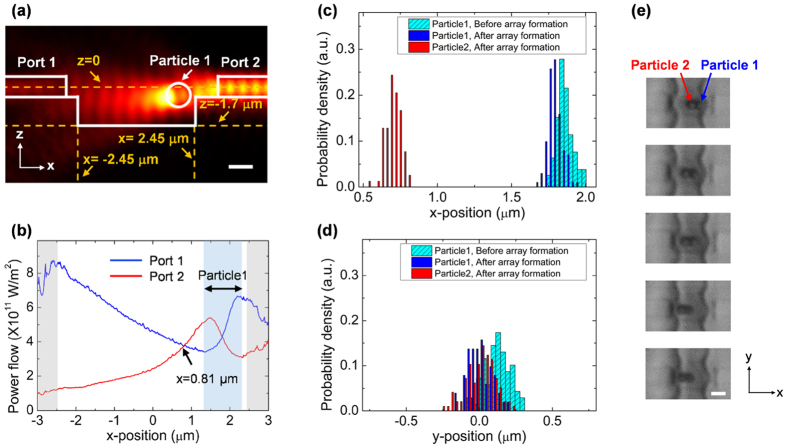
(**a**,**b**) Are 3D Finite element method (FEM) simulations. Note that a trapped particle 1 is located at x = 1.85 μm, y = 0, and z = −375 nm position; (**a**) |*E*|^2^ distribution, cut parallel to the waveguide. A beam is excited on Port 2 and the beam power of 1 W is assumed. Scale bar, 1 μm. (**b**) Calculated power flow of the counter-propagating guided beams. Note that the power ratio of Port 1 to Port 2 is assumed by 2.3. (**c**,**d**) Are longitudinal and transverse positions of trapped particles, as obtained by analyzing the CCD images. (**e**) Time-lapsed images of the trapped particles in the array, showing that the array can be moved across the channel as a single unit. Scale bar, 2 μm.

Counter-propagating beams can produce inter-particle forces mediated by light known as ‘optical binding’^48^. In this section, we describe trapping of multiple particles in a linear array using our trap. A single particle is first trapped between the waveguides, and then an additional particle is trapped next to the first particle, thus forming an array, as shown in Fig. 3. Here we concentrate on analyzing trapping of two particles with a diameter of 1 μm. We note, however, that trapping of more particles with a diameter of 0.65 μm diameter is also possible, as was already shown in Fig. 3f. Figure 6a,b show the calculated E-field distribution and the optical power flow when a single 1 μm diameter particle (Particle1) is trapped at x = 1.85 μm, y = 0, and z = −375 nm position as in Fig. 3b. Using calculated results shown in Fig. 4f, the power ratio in this case is estimated to be 2.3. A second equilibrium position is found to form at x = 0.81 μm, which is 1.04 μm away from the first trapped position. Figure 6c,d show the experimentally obtained position distributions of each trapped particle. Particle 1 moves slightly to the left upon trapping of particle 2 such that the center-to-center distance between two particles is 1.1 ± 0.1 μm. This value agrees well with the calculated value of 1.04 μm shown in Fig. 6b. The array is quite stable, and can be moved across the channel as a single unit by controlling the power ratio, as shown in Fig. 6e.

## Conclusion

We have demonstrated stable, free space optical trapping and manipulation of sub-micron particles in a microfluidic chip using counter-propagating beams compared with numerical simulations. A trench that is designed to be deeper and wider than an optical mode cuts across an inverted waveguide, creating a channel for free-space trapping and enabling full utilization of optical powers. Use of an inverted ridge waveguide structure provides a low propagation loss and prevents unwanted trapping on the waveguide by the evanescent fields, while use of SU8 reduces beam divergence such that stiff trapping with low input power is possible. Both single particle and multiple particle trappings are possible, with the ability to manipulate their longitudinal position by varying the power ratio. For trapping of a single particle of 1 μm diameter, trapping via scattering force with a resolution of < 100 nm is demonstrated with a 10 k_B_T threshold power of less than 1 mW, with a stiffness of 1.35 and 0.12 pN μm^−1^ mW^−1^ for longitudinal and transverse direction for a 1 μm diameter polystyrene particle.

## Methods

### Numerical Simulation Details

3D Simulation is performed based on the finite element method (FEM) using COMSOL 5.1. The wavelength of the guided light is 1550 nm. At this wavelength, refractive indices of SU-8, SiO_2_ and water at 1550 nm are 1.578, 1.444 and 1.318, respectively. A particle is a polystyrene sphere with the diameter of 1 μm and the refractive index of 1.58.

### Experimental Setup

A tunable laser diode and Erbium-doped fiber amplifier (EDFA) are used as an input source. The beam is split, and then input into the SU-8 waveguides. A power attenuator is used to control the relative optical power in each of the counter-propagating beams, and a polarization controller is used to excite the TM mode.

### Imaging and tracking of trapped particles

A trapped particle for each frame in the movie is recorded by CCD camera which allows a full frame size of 1024 × 1024 pixels with a 5.5 μm pixel attached with a 40X objective lens (NA = 0.60). The estimated resolution of the microscope/CCD system is 165 nm per pixel. The objective lens is held on a piezoelectric translation stage to manipulate a focal plane of the microscope along the z-axis. The images are acquired at 70 frames per second (f.p.s.). And a position of the trapped particle is obtained by fitting a circular Gaussian to the camera image^55^. Also, we measure a position of an edge of a waveguide to track the particle position relatively.

### Calculation of trapping force

The electromagnetic force exerting on a particle is expressed as below^53^,


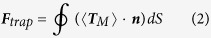


where 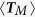 is the time averaged Maxwell Stress Tensor (MST) and ***n*** is the normal vector.

The gradient force ***F***_*trap*_ for Rayleigh particle is described by


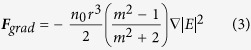


where *n*_0_ is the refractive index of medium, *r* is particle radius and *m* = *n*_1_/*n*_0_ is the relative index of the particle.

## Additional Information

**How to cite this article**: Kim, J. and Shin, J. H. Stable, Free-space Optical Trapping and Manipulation of Sub-micron Particles in an Integrated Microfluidic Chip. *Sci. Rep.* **6**, 33842; doi: 10.1038/srep33842 (2016).

## Supplementary Material

Supplementary Information

Supplementary Movie S1

Supplementary Movie S2

Supplementary Movie S3

Supplementary Movie S4

## Figures and Tables

**Table 1:** Stability, threshold power, and trap stiffness.

Diameter (μm)			Stability number (/1 W at each port)	Threshold power (mW) at each port, U_trap_ = 10 k_B_T	Stiffness (pN/μm/1 mW at each port)
1	x-direction (longitudinal)	Experiment			1.35
	y-direction (transverse)	Simulation (MST)	34153.5	0.29	0.12
		Experiment			0.12
	z-direction (vertical)	Simulation (MST)	41373.8	0.24	0.27
0.65	y-direction	Simulation (MST)	9816.3	1.02	0.040
	z-direction	Simulation (MST)			0.061
0.5	y-direction	Simulation (MST)	5011.8	1.99	0.014
		Simulation (Rayleigh)	4754.4	2.10	0.017
	z-direction	Simulation (MST)			0.029
		Simulation (Rayleigh)			0.029
