# Supplementary material for: Stable, Free-space Optical Trapping and Manipulation of Sub-micron Particles in an Integrated Microfluidic Chip
Source: Sci Rep. 2016 Sep 22;6:33842. doi: 10.1038/srep33842 (PMC5031986; doi:10.1038/srep33842)
Supplement: Supplementary Information [file srep33842-s1.pdf]

Supplementary Information

Stable, Free-space Optical Trapping and Manipulation of Sub-micron Particles in an Integrated Microfluidic Chip

Jisu Kim[1] and Jung H.Shin[2,1]*

[1] KAIST, Department of Physics, 373-1 Guseong-dong, Yuseong-Gu, Daejeon, South Korea
[2] KAIST, Graduate School of Nanoscience and Technology, 373-1 Guseong-dong, Yuseong-Gu, Daejeon, South Korea
*jhs@kaist.ac.kr

Movie 1. Trapping results of 1 μm particles moving along the channel perpendicular to waveguides (defined to be the y-direction).

Movie 2. Single and multiple trapping of 0.65 μm diameter polystyrene particles.

Movie 3 shows particle manipulation, in which we trap a particle at an arbitrary position along the x-dirction between waveguides by simply varying power ratio using external power attenuators.

Supplementary Movie S1. x-component of Poynting vector over time, as calculated by finite-difference time-domain (FDTD) simulation.

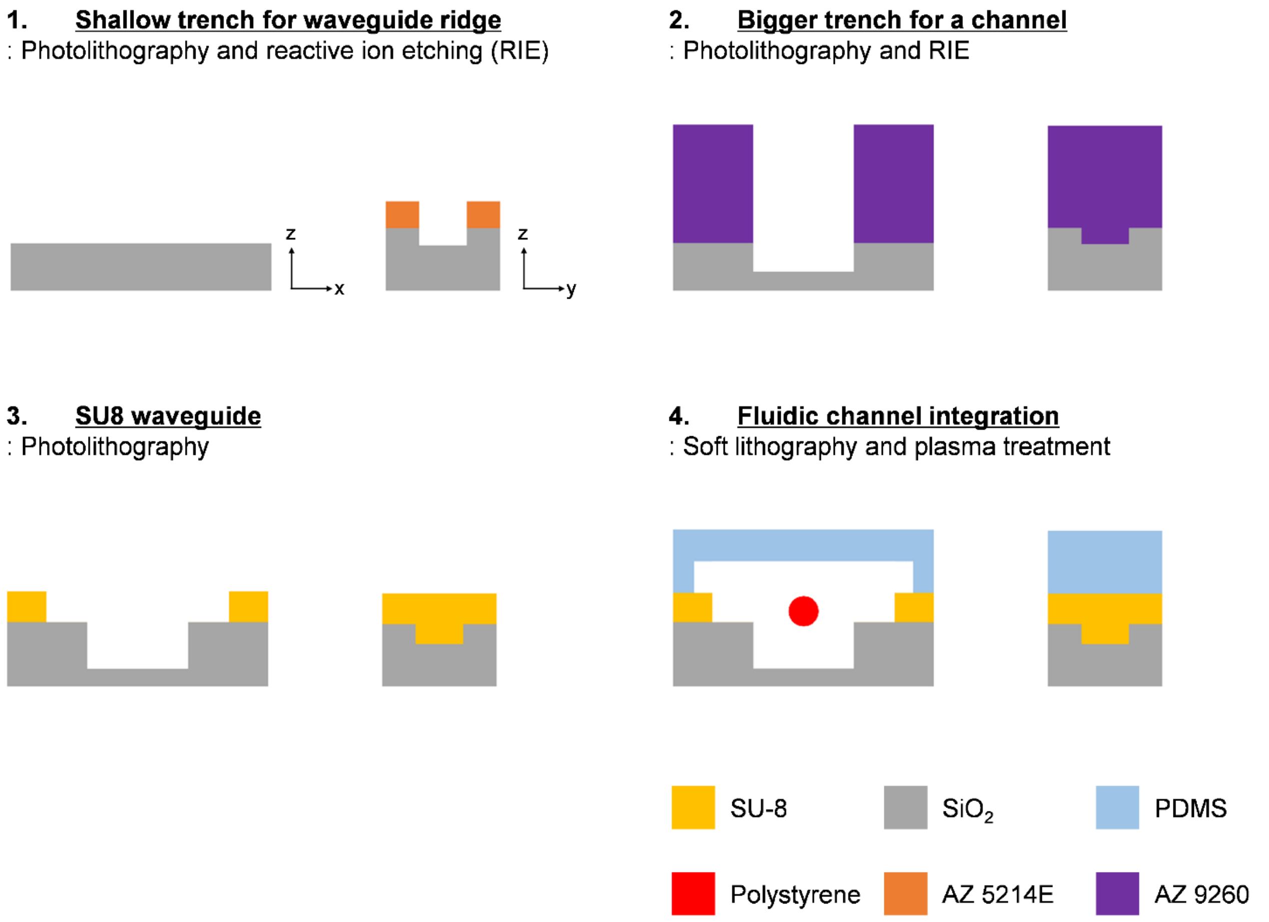

Supplementary Figure S1. A schematic description of the fabrication process in cross-sectional view. Left figures each step are described the xz plane at y=0, cut parallel to a waveguide. Right figures represent the yz plane of the waveguide.

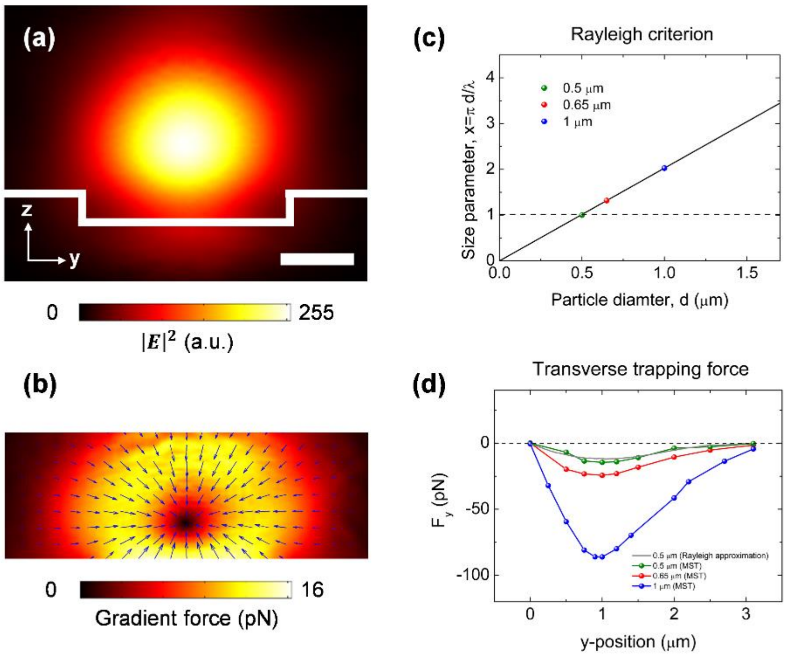

Supplementary Figure S2. 3D Finite element method (FEM) simulations. (a) $|\boldsymbol{E}|^2$ distribution at power equilibrium position (in Fig. 2d). (b) In-plane force and direction on a polystyrene particle with a diameter of 0.5μm assuming it as a Rayleigh particle. (c) Rayleigh criterion. In Rayleigh regime, where a diameter is less than λ/π, optical gradient force is proportional to the volume of particles. Meanwhile, Maxwell Stress Tensor (MST) is used where the diameter is greater than or equal to the value of λ/π . (d) Transverse optical force with different particle sizes. Circle symbols on lines are calculated results using time averaged Maxwell Stress Tensor (MST) and the solid lines are calculated by assuming Rayleigh regime. Scale bar, 1μm.
